# Clinical outcomes by baseline metastases in patients with renal cell carcinoma treated with lenvatinib plus pembrolizumab versus sunitinib: Post hoc analysis of the CLEAR trial

**DOI:** 10.1002/ijc.35288

**Published:** 2024-12-30

**Authors:** Viktor Grünwald, Rana R. McKay, Tomas Buchler, Masatoshi Eto, Se Hoon Park, Toshio Takagi, Sylvie Zanetta, Daniel Keizman, Cristina Suárez, Sylvie Négrier, Jae Lyun Lee, Daniele Santini, Jens Bedke, Michael Staehler, Christian Kollmannsberger, Toni K. Choueiri, Robert J. Motzer, Joseph E. Burgents, Ran Xie, Chinyere E. Okpara, Thomas Powles

**Affiliations:** ^1^ Interdisciplinary Genitourinary Oncology, Clinic for Urology, Clinic for Medical Oncology University Hospital Essen Essen Germany; ^2^ Division of Hematology‐Oncology University of California San Diego La Jolla California USA; ^3^ Department of Oncology, First Faculty of Medicine Charles University and Thomayer University Hospital Prague Czech Republic; ^4^ Department of Urology Kyushu University Fukuoka Japan; ^5^ Division of Hematology/Oncology, Department of Medicine, Samsung Medical Center Sungkyunkwan University School of Medicine Seoul South Korea; ^6^ Department of Urology Tokyo Women's Medical University Tokyo Japan; ^7^ Department of Oncology Georges‐François Leclerc Cancer Centre Dijon France; ^8^ Department of Oncology, Tel‐Aviv Sourasky Medical Center and School of Medicine Tel‐Aviv University Tel‐Aviv Israel; ^9^ Medical Oncology, Vall d'Hebron Institute of Oncology (VHIO) Hospital Universitari Vall d'Hebron, Vall d'Hebron Barcelona Hospital Campus Barcelona Spain; ^10^ University of Lyon, Centre Léon Bérard Lyon France; ^11^ Department of Oncology and Internal Medicine University of Ulsan College of Medicine, Asan Medical Center Seoul South Korea; ^12^ Department of Medical Oncology A, Policlinico Umberto 1 La Sapienza Università di Roma Rome Italy; ^13^ Department of Urology and Transplantation Surgery Eva Mayr‐Stihl Cancer Center, Klinikum Stuttgart Stuttgart Germany; ^14^ Department of Urology University Hospital of Munich Munich Germany; ^15^ Department of Medical Oncology BC Cancer – Vancouver Cancer Centre Vancouver British Columbia Canada; ^16^ Department of Medical Oncology Dana‐Farber Cancer Institute Boston Massachusetts USA; ^17^ Department of Medicine Memorial Sloan Kettering Cancer Center New York New York USA; ^18^ Global Clinical Development, Merck & Co., Inc. Rahway New Jersey USA; ^19^ Biostatistics, Eisai Inc. Nutley New Jersey USA; ^20^ Clinical Research, Eisai Ltd. Hatfield UK; ^21^ Department of Oncology The Royal Free NHS Trust London UK

**Keywords:** pembrolizumab, lenvatinib, lenvatinib plus pembrolizumab, renal cell carcinoma

## Abstract

Lenvatinib plus pembrolizumab significantly improved efficacy versus sunitinib in treatment of advanced renal cell carcinoma (aRCC) in the phase 3 CLEAR study. We report results of an exploratory post hoc analysis of tumor response data based on baseline metastatic characteristics of patients who received lenvatinib plus pembrolizumab versus sunitinib, at the final overall survival analysis time point of CLEAR (cutoff: July 31, 2022). Treatment‐naïve adults with aRCC were randomized to: lenvatinib (20 mg PO QD in 21‐day cycles) plus pembrolizumab (*n* = 355; 200 mg IV Q3W); lenvatinib plus everolimus (not reported here); or sunitinib (*n* = 357; 50 mg PO QD; 4 weeks on/2 weeks off). The most common (lenvatinib plus pembrolizumab; sunitinib, respectively) metastatic site was lung (71.0%; 63.9%), followed by lymph node (45.6%; 43.7%), bone (22.5%; 24.9%), and liver (17.7%; 19.6%). Across treatment arms, ≥65% had two or more metastatic organs/sites involved, >80% of patients had nontarget lesions, and ~45% had baseline sums of diameters of target lesions ≥60 mm. Lenvatinib plus pembrolizumab demonstrated greater progression‐free survival, objective response rate, and duration of response versus sunitinib across evaluable subgroups regardless of site or size of baseline metastasis or number of metastatic sites at baseline. Overall survival generally trended to favor lenvatinib plus pembrolizumab versus sunitinib; and tumor shrinkage was greater across sites (lung, lymph node, liver, and bone) for patients in the lenvatinib‐plus‐pembrolizumab arm versus the sunitinib arm. These results further support lenvatinib plus pembrolizumab as a standard‐of‐care in patients with aRCC regardless of site or size of baseline metastasis or the number of metastatic sites.

## Graphical abstract

What's new?

In the CLEAR trial, lenvatinib plus pembrolizumab showed better efficacy than sunitinib in the treatment of patients with advanced renal cell carcinoma. In this exploratory post hoc subgroup analysis, the authors report a greater progression‐free survival, response rate, and response duration for the drug combination than for sunitinib regardless of the patients' baseline metastatic characteristics. The findings support the use of lenvatinib plus pembrolizumab as a standard‐of‐care in all patients with advanced renal cell carcinoma, regardless of the site or size of baseline metastasis or the number of metastatic sites.

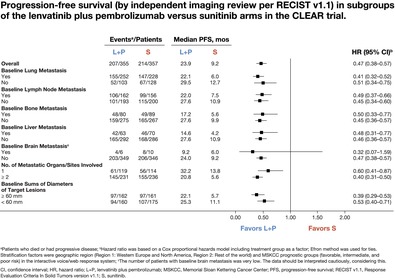

## 1 INTRODUCTION

Clinicopathological features observed in patients with advanced renal cell carcinoma (aRCC), including the site of tumor metastasis, number of metastatic sites, and/or tumor size, may affect the prognosis of the disease.^1^, ^2^, ^3^, ^4^ Per data from an international cohort study of the International Metastatic RCC Database Consortium (IMDC)^1^ that included more than 10,000 patients with metastatic RCC, the most common sites of metastasis in patients were lung (70%), lymph nodes (45%), bone (32%), liver (18%), and brain (8%). Overall survival (OS) varied among patients by sites of metastasis, with metastases to endocrine glands associated with improved survival and metastases to liver or brain associated with shorter OS.^1^ Furthermore, the involvement of multiple metastatic sites corresponded with shorter OS.^2^

The phase 3, multicenter, open‐label, randomized CLEAR trial (Study 307/KEYNOTE‐581) compared the efficacy and safety of lenvatinib plus pembrolizumab versus sunitinib as a first‐line treatment for patients with aRCC.^5^ In the primary analysis of the CLEAR trial (data cutoff: August 28, 2020), with a median survival follow‐up of 26.6 months, lenvatinib plus pembrolizumab demonstrated significantly improved progression‐free survival (PFS: final analysis; hazard ratio [HR] 0.39 [95% CI 0.32–0.49]; *p* < .001) and OS (interim analysis; HR 0.66 [95% CI 0.49–0.88]; *p* = .005) versus sunitinib.^5^ The objective response rate (ORR) also favored lenvatinib plus pembrolizumab (71.0% [95% CI 66.3–75.7]) versus sunitinib (36.1% [95% CI 31.2–41.1]; relative risk 1.97 [95% CI 1.69–2.29]).^5^ Analysis of corresponding data by subgroups of patients at this data cutoff date showed that efficacy outcomes were improved after treatment with lenvatinib plus pembrolizumab versus sunitinib, irrespective of the presence or absence of prognostic indicators of the disease,^1^, ^2^, ^6^, ^7^ including baseline lung metastases, baseline bone metastases, baseline liver metastases, prior nephrectomy, or sarcomatoid features.^8^

At the final prespecified OS analysis, with a median survival follow‐up of approximately 4 years (data cutoff: July 31, 2022), lenvatinib plus pembrolizumab continued to show clinically meaningful efficacy compared with sunitinib in the first‐line treatment of patients with aRCC.^9^ In this exploratory post hoc analysis, we examined outcome data based on the baseline characteristics of the site of metastasis, number of metastatic sites, and baseline sums of diameters of target lesions in patients who received lenvatinib plus pembrolizumab versus sunitinib, at the final OS analysis time point of CLEAR with 23 additional months of follow‐up from the primary analysis. These analyses could potentially inform the choice of personalized therapy in patients with aRCC.

## 2 MATERIALS AND METHODS

### 2.1 Patients and study design

Eligibility criteria for the open‐label, multicenter, randomized CLEAR trial were published previously.^5^ Briefly, treatment‐naïve patients with aRCC that had a clear‐cell component were eligible if they had at least one measurable lesion per Response Evaluation Criteria in Solid Tumors version 1.1 (RECIST v1.1); a Karnofsky performance status score of 70 or higher; and adequate organ function.

Patients were randomly assigned (1:1:1) to receive either lenvatinib 20 mg orally once daily plus pembrolizumab 200 mg intravenously once every 3 weeks, lenvatinib 18 mg plus everolimus 5 mg orally once daily (not reported here), or sunitinib 50 mg orally once daily (4 weeks on/2 weeks off). Randomization was stratified by geographic region (Western Europe and North America or the rest of the world) and by Memorial Sloan Kettering Cancer Center (MSKCC) prognostic risk group (favorable, intermediate, or poor risk).

The primary and key secondary objectives of the trial were met, in which lenvatinib plus pembrolizumab showed statistically significant and/or clinically meaningful improvements in OS, PFS, and ORR (data cutoff date: August 28, 2020).^5^ Data presented here correspond to the data cutoff date of the final prespecified OS analysis (July 31, 2022),^9^ with 23 months of additional follow‐up beyond the primary analysis of CLEAR for a total follow‐up time of ~4 years.^5^ Our analyses focus on the approved combination of lenvatinib plus pembrolizumab versus sunitinib; given the differences in mechanism of action of lenvatinib plus everolimus, related data are not presented here and may be explored later.

#### 2.1.1 Statistics

In this exploratory post hoc analysis, baseline characteristics related to lesion organ/site and non‐target lesions were derived based on independent imaging review. We examined the endpoints of PFS, tumor response, and duration of response (DOR) by independent imaging review per RECIST v1.1 and OS in patients with the following baseline metastatic characteristics: patients with lung, lymph node, bone, liver, or brain metastases; patients with one metastatic site versus those with two or more metastatic sites; and patients with baseline sums of diameters of target lesions ≥60 mm or <60 mm. The cutoff of 60 mm was chosen because the median sums of diameters of target lesions was 60.06 mm in the lenvatinib‐plus‐pembrolizumab arm and 57.96 mm in the sunitinib arm.

Median PFS, OS, and DOR were estimated with the Kaplan–Meier product‐limit method, and 95% CIs were constructed with a generalized Brookmeyer and Crowley method. HR was based on a Cox proportional hazards model stratified by interactive voice/web response system stratification factors (geographic region and MSKCC prognostic groups), including the treatment group as a factor; the Efron method was used for ties. Odds ratios were calculated using the Cochran–Mantel–Haenszel method, stratified by geographic region and MSKCC prognostic groups.

Waterfall plots with the percentage change in sums of diameters of target lesions at nadir by the site of metastasis are also included. These plots include data from patients with baseline and at least one post‐baseline target lesion assessment for the specified site.

## 3 RESULTS

### 3.1 Patients and baseline characteristics

Baseline characteristics of the 1069 patients randomized across treatment arms in the CLEAR trial have been previously described^5^, ^9^ and are summarized in Table S1. Of the randomized patients, 355 were in the lenvatinib‐plus‐pembrolizumab arm, and 357 were in the sunitinib arm. In both treatment arms, patients were similarly distributed across IMDC favorable (lenvatinib plus pembrolizumab, 31.0%; sunitinib, 34.7%), intermediate (59.2% and 53.8%, respectively), and poor risk subgroups (9.3% and 10.4%, respectively) (Table S1). In the lenvatinib‐plus‐pembrolizumab arm, 252 (71%) patients had lung metastases, 162 (45.6%) patients had lymph node metastases, 80 (22.5%) patients had bone metastases, and 63 (17.7%) patients had liver metastases. Correspondingly, in the sunitinib arm, 228 (63.9%) patients had lung metastases, 156 (43.7%) had lymph node metastases, 89 (24.9%) patients had bone metastases, and 70 (19.6%) patients had liver metastases. The number of patients with baseline brain metastasis across treatment arms was very low (≤10); thus, corresponding data should be interpreted with caution.

Across treatment arms, approximately one third of patients had one metastatic organ/site involved, whereas ≥65% had two or more metastatic organs/sites involved (Table S1). More than 80% of patients across treatment arms had non‐target lesions (Table S1). Approximately 45% of patients had ≥60 mm baseline sums of target lesions across treatment arms (Table S1).

### 3.2 Efficacy

#### 3.2.1 Progression‐free survival

**FIGURE 1:**
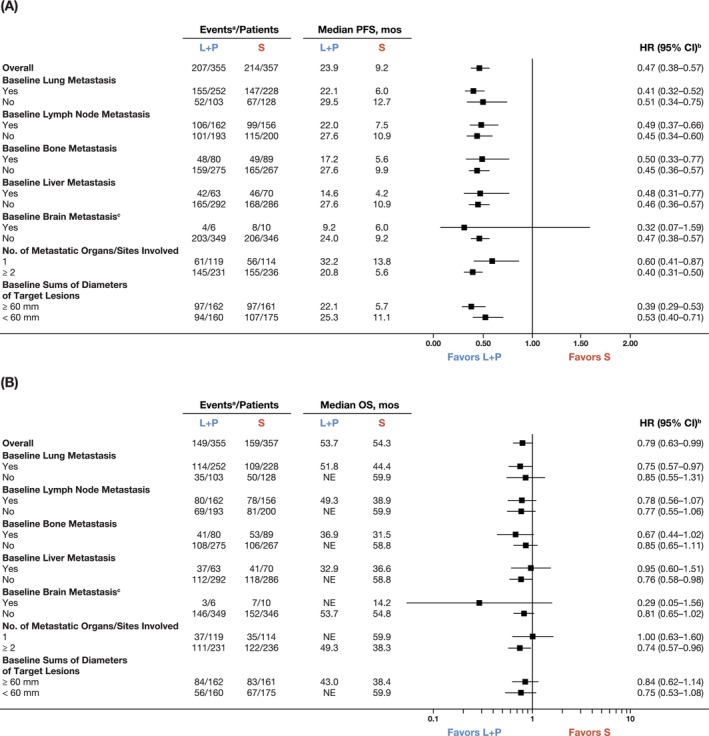
Progression‐free survival by independent imaging review per RECIST v1.1 (A), and overall survival (B) in subgroups of the lenvatinib plus pembrolizumab versus sunitinib arms. Data for all subgroups in (A) were derived based on information obtained from the independent imaging review. Median PFS and OS were estimated with Kaplan–Meier method, and 95% CI was constructed with a generalized Brookmeyer and Crowley method. Stratification factors were geographic region (Region 1: Western Europe and North America, Region 2: Rest of the world) and MSKCC prognostic groups (favorable, intermediate, and poor risk) in the interactive voice/web response system. If a stratification factor was within its own subgroup, this factor was excluded from stratified analysis. ^a^Patients who died or had progressive disease (for PFS); patients who died (for OS); ^b^Hazard ratio was based on a Cox proportional hazards model including treatment group as a factor; Efron method was used for ties. Stratification factors were geographic region (Region 1: Western Europe and North America, Region 2: Rest of the world) and MSKCC prognostic groups (favorable, intermediate, and poor risk) in the interactive voice/web response system; ^c^The number of patients with baseline brain metastasis was very low. The data should be interpreted cautiously, considering this. CI, confidence interval; DOR, duration of response; HR, hazard ratio; L + P, lenvatinib plus pembrolizumab; MSKCC, Memorial Sloan Kettering Cancer Center; NE, not estimable; ORR, objective response rate; OS, overall survival; PFS, progression‐free survival; RECIST v1.1, Response Evaluation Criteria in Solid Tumors version v1.1; S, sunitinib.

The PFS highly favored lenvatinib plus pembrolizumab versus sunitinib across all subgroups with baseline metastasis in the: lung (median 22.1 vs. 6.0 months; HR 0.41 [95% CI 0.32–0.52]), lymph node (median 22.0 vs. 7.5 months; HR 0.49 [95% CI 0.37–0.66]), bone (median 17.2 vs. 5.6 months; HR 0.50 [95% CI 0.33–0.77]), or liver (median 14.6 vs. 4.2 months; HR 0.48 [95% CI 0.31–0.77]) (Figure 1A). Similar results were seen in patients without baseline metastasis in the: lung (median 29.5 vs. 12.7 months; HR 0.51 [95% CI 0.34–0.75]), lymph node (median 27.6 vs. 10.9 months; HR 0.45 [95% CI 0.34–0.60]), bone (median 27.6 vs. 9.9 months; HR 0.45 [95% CI 0.36–0.57]), or liver (median 27.6 vs. 10.9 months; HR 0.46 [95% CI 0.36–0.57]) (Figure 1A). In patients with brain metastases, the CIs for HR were wide (0.32, 95% CI 0.07–1.59), likely due to the small number of patients. Among patients without brain metastases, PFS highly favored the combination versus sunitinib (median 24.0 vs. 9.2 months; HR 0.47 [95% CI 0.38–0.57]) (Figure 1A).

PFS also favored lenvatinib plus pembrolizumab versus sunitinib in patients with one (median 32.2 vs. 13.8 months; HR 0.60 [95% CI 0.41–0.87]) or two or more (median 20.8 vs. 5.6 months; HR 0.40 [95% CI 0.31–0.50]) metastatic organ(s)/site(s) involvement and in patients with baseline sums of diameters of target lesions ≥60 mm (median 22.1 vs. 5.7 months; HR 0.39 [95% CI 0.29–0.53]) or <60 mm (median 25.3 vs. 11.1 months; HR 0.53 [95% CI 0.40–0.71]) (Figure 1A).

#### 3.2.2 Overall survival

The OS trended to favor lenvatinib plus pembrolizumab versus sunitinib in the following subgroups of patients with baseline metastasis in the: lung (median 51.8 vs. 44.4 months; HR 0.75 [95% CI 0.57–0.97]), lymph node (median 49.3 vs. 38.9 months; HR 0.78 [95% CI 0.56–1.07]), or bone (median 36.9 vs. 31.5 months; HR 0.67 [95% CI 0.44–1.02]) (Figure 1B). In patients with liver metastasis, the HR for comparison had wide CIs (HR 0.95 [95% CI 0.60–1.51]), so these data should be interpreted with caution (Figure 1B).

The OS trended to favor lenvatinib plus pembrolizumab versus sunitinib in the following subgroups without baseline metastasis in the: lung (median not estimable vs. 59.9 months; HR 0.85 [95% CI 0.55–1.31]), lymph node (median not estimable vs. 59.9 months; HR 0.77 [95% CI 0.55–1.06]), bone (median not estimable vs. 58.8 months; HR 0.85 [95% CI 0.65–1.11]), or liver (median not estimable vs. 58.8 months; HR 0.76 [95% CI 0.58–0.98]) (Figure 1B). Very few patients had baseline brain metastases; this was reflected by the relatively wide CI for HR (0.29, 95% CI 0.05–1.56). Among patients without brain metastases, the OS trended to favor the combination versus sunitinib (HR 0.81 [95% CI 0.65–1.02]) (Figure 1B).

OS trended to favor lenvatinib plus pembrolizumab versus sunitinib in patients with two or more metastatic organs/sites (median 49.3 vs. 38.3 months; HR 0.74 [95% CI 0.57–0.96]) and in patients with baseline sums of diameters of target lesions ≥60 mm (median 43.0 vs. 38.4 months; HR 0.84 [95% CI 0.62–1.14]) or <60 mm (median not estimable vs. 59.9 months; HR 0.75 [95% CI 0.53–1.08]) (Figure 1B). In patients with one metastatic organ/site involvement, the HR was 1.00 (95% CI 0.63–1.60), but the number of death events were <40 in each arm (Figure 1B), which was also reflected by the relatively wide CI.

#### 3.2.3 Objective response rate and duration of response

**FIGURE 2:**
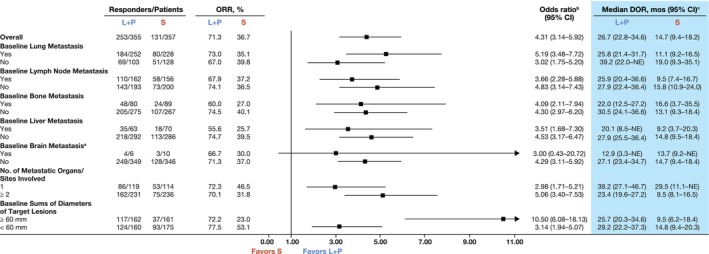
Tumor response and duration of response by independent imaging review per RECIST v1.1 in subgroups of the lenvatinib plus pembrolizumab versus sunitinib arms. ^a^The number of patients with baseline brain metastasis was very low. The data should be interpreted cautiously, considering this; ^b^Odds ratios were calculated using the Cochran–Mantel–Haenszel method, stratified by interactive voice/web response system stratification factors; ^c^Median DOR is for all responders. Medians were estimated with Kaplan–Meier product‐limit method and 95% CIs were constructed with a generalized Brookmeyer and Crowley method. Derivation of data was based on information obtained from the independent imaging review. Stratification factors were geographic region (Region 1: Western Europe and North America, Region 2: Rest of the world) and MSKCC prognostic groups (favorable, intermediate, and poor risk) in the interactive voice/web response system. If a stratification factor was within its own subgroup, this factor was excluded from stratified analysis. Arrows indicate 95% CI values that fall outside the scale of the graph. CI, confidence interval; DOR, duration of response; L + P, lenvatinib plus pembrolizumab; MSKCC, Memorial Sloan Kettering Cancer Center; NE, not estimable; ORR, objective response rate; RECIST v1.1, Response Evaluation Criteria in Solid Tumors version v1.1; S, sunitinib.

ORR highly favored lenvatinib plus pembrolizumab versus sunitinib across all subgroups with baseline metastasis in the: lung (73.0% vs. 35.1%; odds ratio 5.19 [95% CI 3.48–7.72]), lymph node (67.9% vs. 37.2%; odds ratio 3.66 [95% CI 2.28–5.88]), bone (60.0% vs. 27.0%; odds ratio 4.09 [95% CI 2.11–7.94]), or liver (55.6% vs. 25.7%; odds ratio 3.51 [95% CI 1.68–7.30]) (Figure 2). Among the few patients with brain metastases, ORR favored the combination versus sunitinib (66.7% vs. 30.0%; odds ratio 3.00 [95% CI 0.43–20.72]). Additionally, ORR highly favored lenvatinib plus pembrolizumab versus sunitinib across all subgroups without baseline metastasis in the: lung (67.0% vs. 39.8%; odds ratio 3.02 [95% CI 1.75–5.20]), lymph node (74.1% vs. 36.5%; odds ratio 4.83 [95% CI 3.14–7.43]), bone (74.5% vs. 40.1%; odds ratio 4.30 [95% CI 2.97–6.20]), liver (74.7% vs. 39.5%; odds ratio 4.53 [95% CI 3.17–6.47]), or brain (71.3% vs. 37.0%; odds ratio 4.29 [95% CI 3.11–5.92]) (Figure 2).

ORR also highly favored lenvatinib plus pembrolizumab versus sunitinib in patients with one (72.3% vs. 46.5%; odds ratio 2.98 [95% CI 1.71–5.21]) or two or more (70.1% vs. 31.8%; odds ratio 5.06 [95% CI 3.40–7.53]) metastatic organ(s)/site(s) involvement, and in patients with baseline sums of diameters of target lesions ≥60 mm (72.2% vs. 23.0%; odds ratio 10.50 [95% CI 6.08–18.13]) or <60 mm (77.5% vs. 53.1%; odds ratio 3.14 [95% CI 1.94–5.07]) (Figure 2).

Additionally, the DOR favored lenvatinib plus pembrolizumab versus sunitinib across all subgroups with baseline metastasis in the: lung (median 25.8 vs. 11.1 months), lymph node (median 25.9 vs. 9.5 months), bone (median 22.0 vs. 16.6 months), or liver (median 20.1 vs. 9.2 months) (Figure 2). Correspondingly, the DOR favored lenvatinib plus pembrolizumab versus sunitinib across all subgroups without baseline metastasis in the: lung (median 39.2 vs. 19.0 months), lymph node (median 27.9 vs. 15.8 months), bone (median 30.5 vs. 13.1 months), liver (median 27.9 vs. 14.8 months), or brain (median 27.1 vs. 14.7 months) (Figure 2). DOR also favored lenvatinib plus pembrolizumab versus sunitinib in patients with one (median 39.2 vs. 29.5 months) or two or more (median 23.4 vs. 9.5 months) metastatic organ(s)/site(s) involvement and patients with baseline sums of diameters of target lesions ≥60 mm (median 25.7 vs. 9.5 months) or <60 mm (median 29.2 vs. 14.8 months) (Figure 2). In the small subgroup of patients with brain metastases, median DOR was similar with lenvatinib plus pembrolizumab (12.9 months) and sunitinib (13.7 months).

#### 3.2.4 Change in sums of diameters of target lesions

**FIGURE 3:**
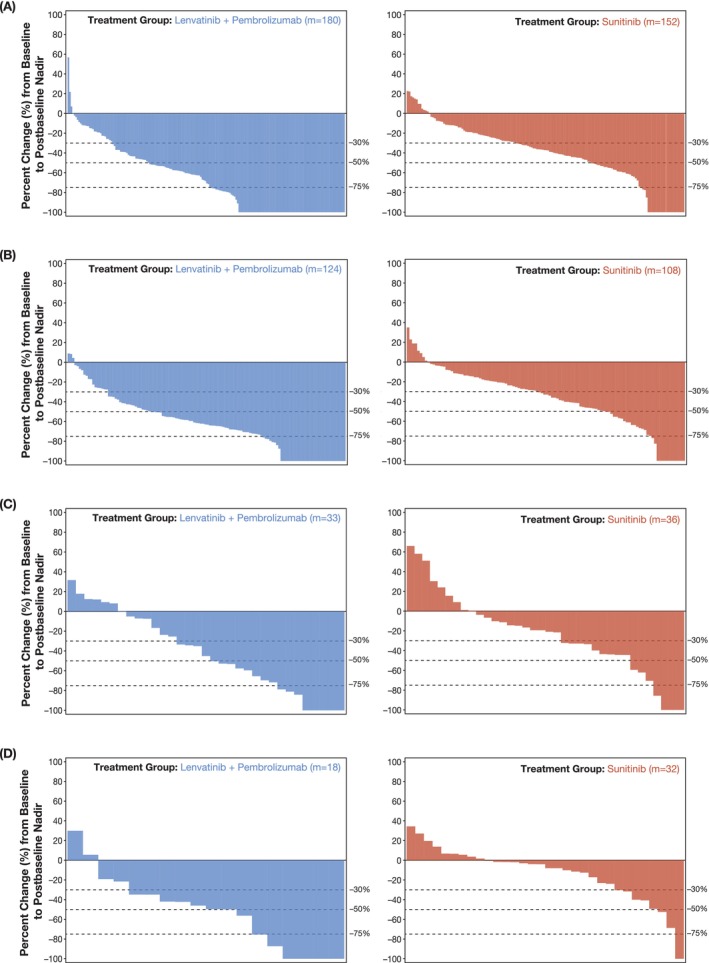
Percentage change in the sum of diameters of target lesions in the lung (A), lymph nodes (B), liver (C), and bone (D) from baseline to postbaseline nadir. These figures include patients (m) with baseline and at least 1 postbaseline target lesion assessment in the respective organ per independent imaging review.

Greater depth and breadth of tumor shrinkage at nadir was observed in target lesions in specific organ sites (lung, lymph node, liver, and bone) for patients in the lenvatinib‐plus‐pembrolizumab arm versus the sunitinib arm (Figure 3). No patients had target lesions in the brain. Patients only had non‐target lesions in the brain.

## 4 DISCUSSION

Patterns of sites of metastatic involvement in aRCC may reflect differences in the underlying disease biology and may affect clinical outcomes,^1^ with metastases to organs like liver, bone, and/or brain typically associated with poor outcomes.^10^, ^11^, ^12^ In this exploratory post hoc subgroup analysis of the CLEAR trial with extended follow‐up of 23 additional months from the primary analysis, OS trended to favor lenvatinib plus pembrolizumab across most subgroups; lenvatinib plus pembrolizumab also demonstrated greater PFS, ORR, and DOR versus sunitinib across evaluable subgroups regardless of site of metastasis (lung, lymph nodes, bones, or liver). These benefits in PFS and tumor response with the combination treatment were also observed regardless of the number of metastatic sites (one or two or more) and baseline sums of diameters of target lesions (≥60 mm or <60 mm). Subgroup analyses were stratified by region and MSKCC prognostic risk groups, consistent with the primary analyses. Results from these subgroups are consistent with outcomes observed in the intent‐to‐treat population at this extended data cutoff date with a median survival follow‐up of ~4 years^9^ and further confirm and supplement the results from the previous exploratory analysis of CLEAR conducted at an earlier data cutoff date.^8^

Although cross‐trial comparisons should be made carefully considering differences in inclusion/exclusion criteria, study design, and methods, subgroup data analysis results from studies in aRCC that used sunitinib as a comparator are briefly summarized for context. In CheckMate‐214 (median follow‐up time of 25.2 months), subgroup OS analysis results favored nivolumab plus ipilimumab over sunitinib across most subgroups, including in patients who did not have bone metastases, in patients irrespective of liver metastases, and in patients with lung metastases.^13^ At the extended follow‐up (median 32.9 months) of CheckMate‐9ER, nivolumab plus cabozantinib showed superior efficacy over sunitinib across subgroups of patients with baseline liver metastasis, with bone metastasis, or with lung metastasis.^14^ In a subgroup analysis of the JAVELIN Renal 101 study that showed improved PFS with avelumab plus axitinib versus sunitinib in the overall population, the observed PFS among patients with baseline brain metastasis (*n* = 23 in each arm) was similar between the two treatment arms, with the HR and median PFS numerically favoring the avelumab arm.^15^ Also, in the phase 3 KEYNOTE‐426 trial, PFS and OS results for subgroups of patients with one or two or more metastatic organs favored pembrolizumab plus axitinib versus sunitinib.^16^ These results emphasize the benefit of immunotherapy‐based combination treatments versus sunitinib in particular prognostic groups. This is in line with observations from our analyses where results of OS, PFS, ORR, and DOR generally favored lenvatinib plus pembrolizumab versus sunitinib across most subgroups of interest. These results emphasize the benefit of combination therapies in treatment of patients with aRCC, compared to previous standard‐of‐care treatments (i.e., sunitinib).

Limitations of presented data include the exploratory nature of our analyses with a lack of statistical power relating to comparisons in individual subgroups and a low number of patients with brain metastases, subgroup data for which should be interpreted with caution. Despite these limitations, the results from these subgroup analyses can provide valuable aid in the selection of personalized treatment strategies for patients and support the use of lenvatinib plus pembrolizumab as a standard‐of‐care treatment across patients with aRCC, regardless of site or size of baseline metastasis or the number of metastatic sites.

In conclusion, lenvatinib plus pembrolizumab showed clinically relevant efficacy in subgroups of interest, including in subgroups based on the site of metastasis, number of metastatic sites, and metastatic tumor size at baseline. Tumor responses favored lenvatinib plus pembrolizumab versus sunitinib in all subgroups of interest. The median DOR was generally longer with lenvatinib plus pembrolizumab versus sunitinib across subgroups of interest. Greater depth and breadth of tumor shrinkage was observed across organ sites (lung, lymph node, liver, and bone) for patients in the lenvatinib‐plus‐pembrolizumab arm versus the sunitinib arm.

Results of this post hoc analysis further support the early, deep, and durable tumor response benefit with lenvatinib plus pembrolizumab versus sunitinib observed in the CLEAR trial^5^, ^9^, ^17^ and support the use of lenvatinib plus pembrolizumab as a standard‐of‐care in all patients with aRCC regardless of site or size of baseline metastasis or the number of metastatic sites.

## AUTHOR CONTRIBUTIONS

**Viktor Grünwald:** Conceptualization; investigation; writing – original draft; writing – review and editing. **Rana R. McKay:** Investigation; writing – original draft; writing – review and editing. **Tomas Buchler:** Investigation; writing – original draft; writing – review and editing. **Masatoshi Eto:** Investigation; writing – original draft; writing – review and editing. **Se Hoon Park:** Investigation; writing – original draft; writing – review and editing. **Toshio Takagi:** Investigation; writing – original draft; writing – review and editing. **Sylvie Zanetta:** Investigation; writing – original draft; writing – review and editing. **Daniel Keizman:** Investigation; writing – original draft; writing – review and editing. **Cristina Suárez:** Investigation; writing – original draft; writing – review and editing. **Sylvie Négrier:** Investigation; writing – original draft; writing – review and editing. **Jae Lyun Lee:** Investigation; writing – original draft; writing – review and editing. **Daniele Santini:** Investigation; writing – original draft; writing – review and editing. **Jens Bedke:** Investigation; writing – original draft; writing – review and editing. **Michael Staehler:** Investigation; writing – original draft; writing – review and editing. **Christian Kollmannsberger:** Investigation; writing – original draft; writing – review and editing. **Toni K. Choueiri:** Investigation; writing – original draft; writing – review and editing. **Robert J. Motzer:** Investigation; writing – original draft; writing – review and editing. **Joseph E. Burgents:** Writing – original draft; writing – review and editing. **Ran Xie:** Conceptualization; formal analysis; writing – original draft; writing – review and editing. **Chinyere E. Okpara:** Conceptualization; writing – original draft; writing – review and editing. **Thomas Powles:** Investigation; writing – original draft; writing – review and editing.

## FUNDING INFORMATION

This study was sponsored by Eisai Inc., Nutley, NJ, USA, and Merck Sharp & Dohme LLC, a subsidiary of Merck & Co., Inc., Rahway, NJ, USA. Medical writing support was funded by Eisai Inc., Nutley, NJ, USA, and Merck Sharp & Dohme LLC, a subsidiary of Merck & Co., Inc., Rahway, NJ, USA.

## CONFLICT OF INTEREST STATEMENT

Viktor Grünwald: Grants/research support from BMS, Ipsen, MSD Oncology, AstraZeneca; honoraria or consultation fees from AAA/Novartis, Amgen, Apogepha, Astellas Pharma, AstraZeneca, BMS, Cureteq, Debiopharm, Eisai, Gilead Sciences, Ipsen, Janssen‐Cilag, Merck Serono, MSD Oncology, Novartis, Oncorena, Ono Pharmaceutical, PCI Biotech, Pfizer, Synthekine; travel support from AstraZeneca, Ipsen, Janssen, Merck Serono, Pfizer. Rana R. McKay: Consultant/advisor for Ambrx, Arcus, AstraZeneca, Aveo, Bayer, Blue Earth Diagnostics, BMS, Calithera, Caris, Daiichi Sankyo, Dendreon, Exelixis, Johnson & Johnson, Lilly, Merck, Myovant, Neomorph, Novartis, Pfizer, Sanofi, SeaGen, Sorrento Therapeutics, Telix, Tempus; institutional research funding from Artera AI, AstraZeneca, Bayer, BMS, Exelixis, Oncternal, Tempus. Tomas Buchler: Invited speaker for Roche, BMS, Astellas, Janssen, Ipsen, Merck, Bayer, Exelixis, Eisai, Eli Lilly, MSD; advisory board for Bayer, BMS, Ipsen, Merck, Servier, Eli Lilly, Pfizer, Accord, AstraZeneca. Non‐financial interests: advisory role for Leram Pharmaceuticals. Masatoshi Eto: Advisory board for Eisai, Chugai Pharmaceutical, Intuitive Surgical, Johnson & Johnson, Merck Biopharma, MSD, Pfizer, Takeda; speaker's bureau for Astellas Pharma, AstraZeneca, Bayer Yakuhin, BMS, Eisai, Janssen Pharmaceutical, Merck Biopharma, MSD, Ono Pharmaceutical, Pfizer, Takeda; research grants from BMS, MSD, Ono Pharmaceutical, Takeda. Se Hoon Park: None. Toshio Takagi: Invited speaker for Eisai. Sylvie Zanetta: None. Daniel Keizman: Invited speaker for MSD, BMS, Pfizer, Astellas; advisory board for MSD, BMS, Pfizer, Astellas, Eisai. Cristina Suárez: Invited speaker for Astellas Pharma, BMS (Inst), Ipsen, Pfizer S.L.U, Hoffmann‐La Roche LTD, Merck; advisory board for Astellas Pharma, Bayer, BMS (Inst), Ipsen, Pfizer S.L.U, Sanofi‐Aventis, Hoffmann‐La Roche LTD, Merck Sharp and Dohme; funding from Ipsen. Sylvie Négrier: Advisory board for Pfizer, BMS, Ipsen, MSD, Eisai; research grants from Pfizer, Ipsen; travel support from Pfizer, Ipsen, MSD, BMS, Eisai. Jae Lyun Lee: Invited speaker for Pfizer, Janssen, Novartis, BMS, Genentech, Roche, AstraZeneca, MSD, Merck, Bayer Schering Pharma, Seagen, GI Innovation, Amgen, Oscotec; advisory board for BMS, Astellas Korea, AstraZeneca, MSD, Merck; stocks/shares from Merck, Johnson and Johnson, Amgen, Black Diamond Therapeutics, Zymeworks, Karyopharm Therapeutics. Daniele Santini: None. Jens Bedke: Invited speaker for Apogepha, AstraZeneca, Astellas, BMS, Eisai, MSD, Ipsen, Novartis, Pfizer, Roche, Seagen; advisory board for AstraZeneca, Astellas, Eisai, BMS, Pfizer, Gilead; advisory board and speaker's bureau from BMS, Ipsen, MSD, Merck Serono, Pfizer, Roche, Janssen; member of the Renal Cell Carcinoma Guidelines Panel: European Association of Urology. Michael Staehler: Consultancy fees and honoraria from Pfizer, GSK, Novartis, Bayer, Aveo, EUSA Pharm, Astellas, Ipsen, Exelixis, Peloton, Eisai, BMS, MSD, Apogepha, Roche, Oncorena, Janssen; research grants from Pfizer, GSK, Aveo, BMS, Novartis, Bayer, Roche/Genentech, Immatics, Wilex, Ipsen, Exelixis, Eisai. Christian Kollmannsberger: Invited speaker for Pfizer, Ipsen, BMS, Merck, Eisai, Astellas, Bayer, Janssen, Seagen; advisory board for Pfizer, Ipsen, BMS, Merck, Eisai, Astellas, Bayer, Janssen, Seagen, BionTech. Non‐financial Interests: principal investigator for BMS, Ipsen, Eisai; advisory board for Merck. Toni K. Choueiri: Reports institutional and/or personal, paid and/or unpaid support for research, advisory boards, consultancy, and/or honoraria past 5 years, ongoing or not, from Alkermes, Arcus Bio, AstraZeneca, Aravive, Aveo, Bayer, BMS, Bicycle Therapeutics, Calithera, Circle Pharma, Deciphera Pharmaceuticals, Eisai, EMD Serono, Exelixis, GSK, Gilead, HiberCell, IQVA, Infinity, Institut Servier, Ipsen, Jansen, Kanaph, Lilly, Merck, Nikang, Neomorph, Nuscan/PrecedeBio, Novartis, Oncohost, Pfizer, Roche, Sanofi/Aventis, Scholar Rock, Surface Oncology, Takeda, Tempest, Up‐To‐Date, CME events (Peerview, OncLive, MJH, CCO and others), outside the submitted work; institutional patents filed on molecular alterations and immunotherapy response/toxicity, and ctDNA; equity in Tempest, Pionyr, Osel, Precede Bio, CureResponse, InnDura Therapeutics, Primium, Bicycle; committees for NCCN, GU Steering Committee, ASCO (BOD 6‐2024), ESMO, ACCRU, KidneyCan; medical writing and editorial assistance support may have been funded by communications companies in part; no speaker's bureau; mentored several non‐US citizens on research projects with potential funding (in part) from non‐US sources/Foreign Components; the institution (Dana‐Farber Cancer Institute) may have received additional independent funding of drug companies or/and royalties potentially involved in research around the subject matter. T. K. Choueiri is supported in part by the Dana‐Farber/Harvard Cancer Center Kidney SPORE (2P50CA101942‐16) and Program 5P30CA006516‐56, the Kohlberg Chair at Harvard Medical School and the Trust Family, Michael Brigham, Pan Mass Challenge, Hinda and Arthur Marcus Fund and Loker Pinard Funds for Kidney Cancer Research at DFCI. Robert J. Motzer: Research funding (paid to institution) from BMS, Eisai, Exelixis, Genentech/Roche, Merck, Pfizer, Aveo Pharmaceuticals; consulting fees from AstraZeneca, Aveo Pharmaceuticals, Eisai, EMD Serono, Exelixis, Genentech/Roche, Incyte, Lilly, Merck, Novartis, Pfizer, Takeda. Joseph E. Burgents: Employee of Merck Sharp & Dohme LLC, a subsidiary of Merck & Co., Inc., Rahway, NJ, USA. Ran Xie: Previous employee of Eisai Inc. Chinyere E. Okpara: Employee of Eisai Ltd. Thomas Powles: Research funding from Astellas Pharma, AstraZeneca, BMS, Eisai, Exelixis, Ipsen, Johnson & Johnson, Merck, Merck Serono, MSD, Novartis, Pfizer, Roche, Seattle Genetics; consulting fees from Astellas Pharma, AstraZeneca, BMS, Eisai, Exelixis, Incyte, Ipsen, Johnson & Johnson, Merck, Merck Serono, MSD, Novartis, Pfizer, Roche, Seattle Genetics; support for attending meetings or travel from AstraZeneca, Ipsen, MSD, Pfizer, Roche.

## ETHICS STATEMENT

The CLEAR trial was conducted in accordance with the International Council for Harmonisation Good Clinical Practice Guidelines and the principles of the 2013 Declaration of Helsinki. The protocol and related documents were approved by Institutional Review Boards or independent ethics committees. All patients provided written informed consent. Efficacy and safety data were monitored by an independent data and safety monitoring committee. ClinicalTrials.gov registration ID: NCT02811861.

## Supporting information


**Table S1:** Baseline demographic and disease characteristics of patients in the CLEAR trial.

## Data Availability

To access the datasets, contact Eisai Inc. Further information, models, and the code that support the findings of this study are available from the corresponding author upon reasonable request.

## References

[1] Dudani S , de Velasco G , Wells JC , et al. Evaluation of clear cell, papillary, and chromophobe renal cell carcinoma metastasis sites and association with survival. JAMA Netw Open. 2021;4:e2021869. 33475752 10.1001/jamanetworkopen.2020.21869 PMC7821027

[2] Wei H , Miao J , Cui J , et al. The prognosis and clinicopathological features of different distant metastases patterns in renal cell carcinoma: analysis based on the SEER database. Sci Rep. 2021;11:17822. 34497343 10.1038/s41598-021-97365-6 PMC8426479

[3] Czarnecka AM , Brodziak A , Sobczuk P , et al. Metastatic tumor burden and loci as predictors of first line sunitinib treatment efficacy in patients with renal cell carcinoma. Sci Rep. 2019;9:7754. 31123336 10.1038/s41598-019-44226-y PMC6533291

[4] DiNatale RG , Xie W , Becerra MF , et al. The association between small primary tumor size and prognosis in metastatic renal cell carcinoma: insights from two independent cohorts of patients who underwent cytoreductive nephrectomy. Eur Urol Oncol. 2020;3:47‐56. 31735646 10.1016/j.euo.2019.10.002 PMC7236081

[5] Motzer R , Alekseev B , Rha SY , et al. Lenvatinib plus pembrolizumab or everolimus for advanced renal cell carcinoma. N Engl J Med. 2021;384:1289‐1300. 33616314 10.1056/NEJMoa2035716

[6] Hahn AW , Lebenthal J , Genovese G , Sircar K , Tannir NM , Msaouel P . The significance of sarcomatoid and rhabdoid dedifferentiation in renal cell carcinoma. Cancer Treat Res Commun. 2022;33:100640. 36174377 10.1016/j.ctarc.2022.100640

[7] Kim SH , Jeong KC , Joung JY , Seo HK , Lee KH , Chung J . Prognostic significance of nephrectomy in metastatic renal cell carcinoma treated with systemic cytokine or targeted therapy: a 16‐year retrospective analysis. Sci Rep. 2018;8:2974. 29445167 10.1038/s41598-018-20822-2 PMC5813006

[8] Grünwald V , Powles T , Eto M , et al. Phase 3 CLEAR study in patients with advanced renal cell carcinoma: outcomes in subgroups for the lenvatinib‐plus‐pembrolizumab and sunitinib arms. Front Oncol. 2023;13:1223282. 37664025 10.3389/fonc.2023.1223282 PMC10471185

[9] Motzer RJ , Porta C , Eto M , et al. Lenvatinib plus pembrolizumab versus sunitinib in first‐line treatment of advanced renal cell carcinoma: final prespecified overall survival analysis of CLEAR, a phase III study. J Clin Oncol. 2024;42:1222‐1228. 38227898 10.1200/JCO.23.01569 PMC11095851

[10] Gong J , Maia MC , Dizman N , Govindarajan A , Pal SK . Metastasis in renal cell carcinoma: biology and implications for therapy. Asian J Urol. 2016;3:286‐292. 29264197 10.1016/j.ajur.2016.08.006 PMC5730828

[11] Chandrasekar T , Klaassen Z , Goldberg H , Kulkarni GS , Hamilton RJ , Fleshner NE . Metastatic renal cell carcinoma: patterns and predictors of metastases—a contemporary population‐based series. Urol Oncol. 2017;35(661):e7‐661.e14. 10.1016/j.urolonc.2017.06.060 28728748

[12] Beuselinck B , Oudard S , Rixe O , et al. Negative impact of bone metastasis on outcome in clear‐cell renal cell carcinoma treated with sunitinib. Ann Oncol. 2011;22:794‐800. 20937648 10.1093/annonc/mdq554

[13] Motzer RJ , Tannir NM , McDermott DF , et al. Nivolumab plus ipilimumab versus sunitinib in advanced renal‐cell carcinoma. N Engl J Med. 2018;378:1277‐1290. 29562145 10.1056/NEJMoa1712126 PMC5972549

[14] Motzer RJ , Powles T , Burotto M , et al. Nivolumab plus cabozantinib versus sunitinib in first‐line treatment for advanced renal cell carcinoma (CheckMate 9ER): long‐term follow‐up results from an open‐label, randomised, phase 3 trial. Lancet Oncol. 2022;23:888‐898. 35688173 10.1016/S1470-2045(22)00290-X PMC10305087

[15] Jonasch E , Hasanov E , Motzer RJ , et al. Evaluation of brain metastasis in JAVELIN Renal 101: efficacy of avelumab + axitinib (A+Ax) versus sunitinib (S) [abstract]. J Clin Oncol. 2020;38(6 Suppl):Abstract 687.

[16] Rini BI , Plimack ER , Stus V , et al. Pembrolizumab plus axitinib versus sunitinib for advanced renal‐cell carcinoma. N Engl J Med. 2019;380:1116‐1127. 30779529 10.1056/NEJMoa1816714

[17] Motzer RJ , Powles T , Hutson T , et al. Characterization of tumor response with lenvatinib plus pembrolizumab in patients with advanced renal cell carcinoma: final overall survival analysis of the CLEAR study (4‐year median follow up). Poster presented at: Kidney Cancer Research Summit (KCRS); July 13–14, 2023; Boston, MA, USA.

