# Supplementary material for: Clinical outcomes by baseline metastases in patients with renal cell carcinoma treated with lenvatinib plus pembrolizumab versus sunitinib: Post hoc analysis of the CLEAR trial
Source: Int J Cancer. 2024 Dec 30;156(7):1326–35. doi: 10.1002/ijc.35288 (PMC11789451; doi:10.1002/ijc.35288)
Supplement: Supplementary file 1 — Table S1: Baseline demographic and disease characteristics of patients in the CLEAR trial. [file IJC-156-1326-s001.pdf]

Supplementary Material

Clinical outcomes by baseline metastases in patients with renal cell carcinoma treated with lenvatinib plus pembrolizumab versus sunitinib: post hoc analysis of the CLEAR trial

Viktor Grünwald, Rana R. McKay, Tomas Buchler, Masatoshi Eto, Se Hoon Park, Toshio Takagi, Sylvie Zanetta, Daniel Keizman, Cristina Suárez, Sylvie Négrier, Jae Lyun Lee, Daniele Santini, Jens Bedke, Michael Staehler, Christian Kollmannsberger, Toni K. Choueiri, Robert J. Motzer, Joseph E. Burgents, Ran Xie, Chinyere E. Okpara, Thomas Powles

Table of contents:

Supplementary Table 1

Supplementary Table S1. Baseline demographic and disease characteristics of patients in the CLEAR trial

Parameter	Treatment group: Lenvatinib + pembrolizumab (n = 355)	Treatment group: Sunitinib (n = 357)
**Age, median (years)**	64 (34-88)	61 (29-82)
**Geographic region, n (%)**		
Western Europe plus North America	198 (55.8)	199 (55.7)
Rest of the world	157 (44.2)	158 (44.3)
**MSKCC prognostic group at baseline,[a] n (%)**		
Favorable risk	96 (27.0)	97 (27.2)
Intermediate risk	227 (63.9)	228 (63.9)
Poor risk	32 (9.0)	32 (9.0)
**IMDC risk group at baseline,[b] n (%)**		
Favorable risk	110 (31.0)	124 (34.7)
Intermediate risk	210 (59.2)	192 (53.8)
Poor risk	33 (9.3)	37 (10.4)
Not evaluable	2 (0.6)	4 (1.1)
**Lesion organ/site location,[c,d] n (%)**		
Lung	252 (71.0)	228 (63.9)
Lymph node	162 (45.6)	156 (43.7)
Bone	80 (22.5)	89 (24.9)
Liver	63 (17.7)	70 (19.6)
Brain	6 (1.7)	10 (2.8)

Parameter	Treatment group	
	Lenvatinib + pembrolizumab (n = 355)	Sunitinib (n = 357)
**Number of metastatic organs/sites involved,[d,e] n (%)**		
0	5 (1.4)	6 (1.7)
1	119 (33.5)	114 (31.9)
≥ 2	231 (65.1)	236 (66.1)
Missing	0 (0.0)	1 (0.3)
**No. of patients with non-target lesions,[d] n (%)**	306 (86.2)	304 (85.2)
**Baseline sum of diameters of target lesions, n (%)**		
≥ 60 mm	162 (45.6)	161 (45.1)
< 60 mm	160 (45.1)	175 (49.0)

One patient in the lenvatinib-plus-pembrolizumab arm had carcinoma without a clear-cell component.

[a]An MSKCC score of 0 indicates favorable risk, a score of 1 or 2 indicates intermediate risk, and a score of 3 or higher indicates poor risk; [b]An IMDC score of 0 indicates favorable risk, a score of 1 or 2 indicates intermediate risk, and a score of 3 to 6 indicates poor risk. IMDC risk group was not a stratification factor and relevant data were derived programmatically; [c]Patients may be represented in > 1 category; [d]Derivation based on information obtained from independent imaging review; [e]Kidney was not included in the number of metastatic organs/sites.

IMDC, International Metastatic RCC Database Consortium; MSKCC, Memorial Sloan Kettering Cancer Center.
